# The Effect of Low and Moderate Exercise on Hyperuricemia: Protocol for a Randomized Controlled Study

**DOI:** 10.3389/fendo.2021.716802

**Published:** 2021-09-02

**Authors:** Yuning Hou, Renyan Ma, Song Gao, Keneilwe Kenny Kaudimba, Hongmei Yan, Tiemin Liu, Ru Wang

**Affiliations:** ^1^School of Kinesiology, Shanghai University of Sport, Shanghai, China; ^2^Department of Endocrinology and Metabolism, Zhongshan Hospital, Fudan Institute for Metabolic Disease, Fudan University, Shanghai, China; ^3^State Key Laboratory of Genetic Engineering, Department of Endocrinology and Metabolism, School of Life Sciences, Institute of Metabolism and Integrative Biology, Human Phenome Institute, Zhongshan Hospital, Fudan University, Shanghai, China

**Keywords:** hyperuricemia, jogging, brisk walking, randomized controlled trial, protocol

## Abstract

**Background:**

Hyperuricemia (HUA) is a metabolic disease by purine metabolism disorders. It is a risk factor for many chronic diseases, including diabetes, hypertension, and heart disease. Studies have shown that exercise can effectively reduce serum uric acid (SUA), but the optimal exercise dose, intensity, and mode of exercise for improving HUA have not been verified in clinical studies. Therefore, this study aims to explore the effect of different exercise intensities in improving SUA of patients with HUA.

**Methods and Analysis:**

A randomized, single-blind, parallel controlled trial will be conducted in this study. 186 HUA patients who meet the inclusion criteria will be randomly divided into a 1:1:1 ratio (1): control group (2), low-intensity exercise group (brisk walking, 57-63% maximum heart rate, 150 min/week, 12 months), and (3) moderate-intensity exercise group (jogging, 64-76% maximum heart rate, 150 min/week, 12 months). The three groups of subjects will receive the same health education and prohibition of high-purine diet during the intervention period. The primary outcomes will be SUA concentration, SUA concentration change (mg/dL), SUA change rate (%), and the proportion of HUA patients. Secondary outcomes will include anthropometric parameters (body weight, waist circumference, hip circumference, BMI); physiological indicators (blood pressure, grip, vital capacity, maximum oxygen); biochemical indicators (blood lipid, blood sugar, liver enzyme, creatinine, and blood urea nitrogen). Each group of patients will go through an assessment at baseline, 3rd, 6th, and 12th months.

**Discussion:**

This study will evaluate the effect of 12-month low-intensity exercise and moderate-intensity exercise on HUA patients. We hypothesize that both low-intensity and moderate-intensity exercise would improve HUA as compared with no-exercise control, and that moderate-intensity exercise would be more effective than low-intensity exercise in improving HUA. These results can provide a basis for the current physical activity guidelines for HUA’s healthy lifestyle management.

**Ethics and Dissemination:**

This study has been approved by the Ethical Review Committee of the Shanghai University of Sport (approval number: 102772020RT005). Informed consent will be obtained from all participants or their guardians. The authors intend to submit the study findings to peer-reviewed journals or academic conferences to be published.

**Clinical Trial Registration:**

Chinese Clinical Trial Registry, identifier ChiCTR2100042643.

## Background

Hyperuricemia (HUA) is a metabolic disease caused by the disorder of purine metabolism, which results in increased production or decreased excretion of serum uric acid (SUA) (1). HUA is defined as under a normal purine diet, the SUA level of two fasting tests on different days, male>7mg/dL, female>6mg/dL (2–4). According to statistics, the number of HUA in China has reached 170 million, and the overall prevalence rate is 13.3% (5). In recent years, the incidence of HUA is on the rise, showing a trend in younger age (5). HUA patients aggravate with the increase of SUA levels, often leading to the occurrence of gout. At present, there are more than 80 million gout patients in China, and it is increasing rapidly at an annual growth rate of 9.7% (5). In addition, HUA is a risk factor for many chronic diseases. When the SUA level increases by 60 μmol/L, the incidence of diabetes increases by 17% (6), the incidence of hypertension increases by 13% (7), and the risk of death from coronary heart disease increases by 12% (8).

The symptoms of HUA are not obvious, which cause delay in treatment. The current management of HUA mainly includes medication and lifestyle adjustments. Xanthine oxidase inhibitors are often used as first-line drugs for HUA, including allopurinol (9) and febuxostat (10, 11). However, the side effects caused by long-term drug use, such as severe skin reactions, renal toxicity, need to be paid attention to (12–14). In addition, HUA patients can reduce SUA by adjusting lifestyles. Studies have shown that a healthy lifestyle including dietary regulation, restriction of tobacco and alcohol, regular exercise, and weight control can reduce SUA concentration by 10%-18% or 70-90mol/L (15–17). A multi-center longitudinal study (18) showed that HUA patients who do not exercise for (<1 hour/week) their mortality increases by 27%, of which life expectancy is shortened by 4.3 years in male, and 5.7 years in female. While HUA patients who exercise for (≥7.5MET/hour/week) mortality reduces by 11%, hence life expectancy is extended by 4-6 years, which shows that adequate exercise can reduce the risk of death and extend life. In addition, a Korean cohort study (4) showed that subjects who were sedentary for ≥10 hours a day were more likely to develop HUA than subjects who were sedentary for <5 hours a day. However, several small clinical trials have found that physical exercise does not have a positive effect on SUA levels (19–22). At present, there is no uniform standard for exercise dose and intensity to prevent or treat HUA (23–25). In addition, the long-term effects of exercise therapy have not been confirmed, and need to be verified in future clinical studies.

In this study, our purpose is to explore the effect of different exercise intensities in improving SUA of patients with HUA. We will conduct a 12-month randomized controlled trial to determine the impact of low-intensity exercise and moderate-intensity exercise on HUA. We hypothesize that both low-intensity and moderate-intensity exercise can improve HUA, and that moderate-intensity exercise is more effective than low-intensity exercise in improving HUA.

## Materials And Methods

### Research Design

**Figure 1:**
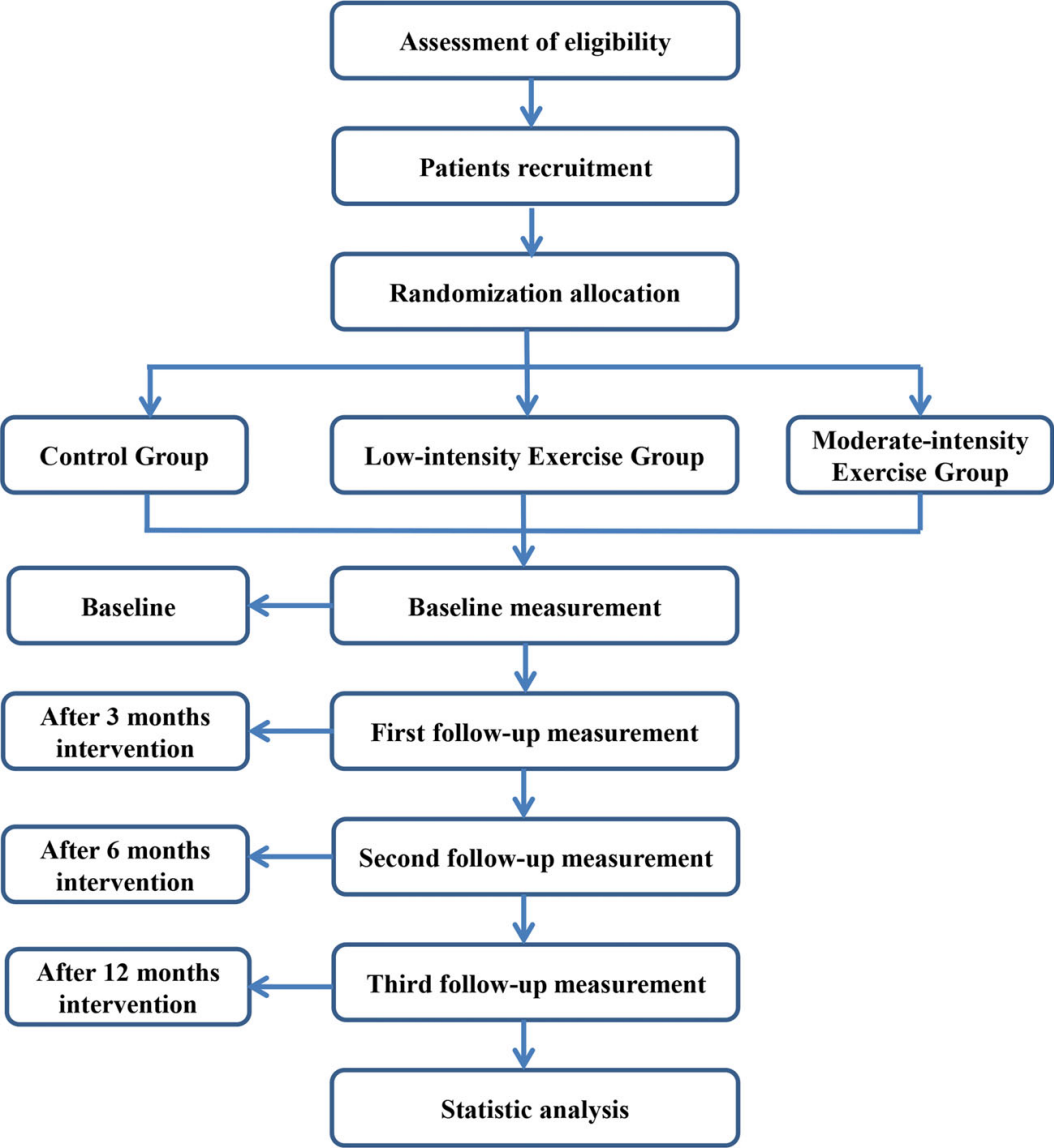
Trail flow chart.

This study is a single-blind, multi-center, parallel randomized controlled trial. Recruitment starts in July 2021 and ends in July 2022. The study period (including the one-year recruitment period) is 3 years. Participants will be randomly divided into a control group, a low-intensity exercise group, and a moderate-intensity exercise group. Tests will be performed at baseline, 3rd, 6th, and 12th months. The research flowchart is shown in **Figure 1**. This study has been ethically approved by the Ethical Review Committee of Shanghai University of Sport (approval number: 102772020RT005, Chinese clinical trial registration: ChiCTR2100042643). All patients should provide written informed consent before enrollment. The study will be following the Guidelines of Comprehensive Reporting Test Standard (CONSORT) (26).

### Research Setting

This study will recruit 186 eligible subjects from multiple medical institutions, such as Zhongshan Hospital with Fudan University, Huashan Hospital Affiliated with Fudan University, Yangpu District Shidong Hospital, Yinhang Community Health Service Center. All research diagnosis and blood index tests will be carried out in the Zhongshan Hospital with Fudan University. Exercise intervention, anthropometric parameters and physiological indicators will be completed in Shanghai University of Sport.

### Inclusion Criteria

(1) Men and women;
(2) Age: 45-60 years old;
(3) Diagnose with HUA, within 4 weeks. Under normal purine diet, two fasting tests on different days, SUA>7mg/dL (male), SUA>6mg/dL(female);
(4) No history of gout;
(5) Agree and sign the informed consent form.

### Exclusion Criteria

(1) Use drugs that may affect uric acid within 1 month before enrollment, including benzbromarone, allopurinol, febuxostat, etc;
(2) Psychiatric conditions;
(3) Serious medical diseases, such as cancer, angina pectoris, cerebral infarction;
(4) Participated in other clinical trials in the past 4 weeks.

### Randomized Allocation and Blinding

After the baseline assessment, the study will use a 1:1:1 ratio to randomly divide the subjects into 3 groups. The random table will be provided by the Statistics Department of the School of Public Health, Fudan University (Excel software; Microsoft). The test will be completed by unrelated personnel, and the random table will be kept by the main investigator. The researcher will conceal the distribution in an opaque envelope, which can only be opened after the subject has completed the baseline assessment. At the same time, this study is a single-blind randomized trial. In this study, all subjects will be unaware of the allocation of the control group, low-intensity exercise group, and moderate-intensity exercise group. The concentration of uric acid in serum and urine will be measured in Zhongshan Hospital affiliated with Fudan University. The measured values of SUA will be hidden from the investigator and patient to maintain a blind assignment.

### Sample Size Calculation

According to the existing literature (27), after 45 days of short-term moderate-intensity aerobic exercise intervention in patients with HUA, the average improvement value of SUA was 48.8 µmol/L (0.82 mg/dL). This study will carry out a long-term exercise intervention (12 months) in 3 groups. Test power (1-β) is set at 0.80; significance level (α) is set at 0.05. The sample size is estimated using the F test in G*Power (version 3.1.2, Franz Paul, Universit Kiel, Germany). After calculation, the total sample size is 159 cases, with 53 cases in each group. Taking into account the loss to follow-up, the total sample size in the actual study requires 186 subjects based on a 15% loss to follow-up rate. Therefore, at least 62 subjects are included in each group.

### Intervention

#### Control Group

The subjects in the control group will be given health education and maintain their original lifestyle. Control subjects will be given a monthly uric acid popular science lecture, such as the relationship between hyperuricemia and gout, diet control of hyperuricemia patients, and correction of poor lifestyles. During the study period, high-purine diets will be banned.

#### Low-Intensity Exercise Group

Participants in the low-intensity exercise group will be intervened at Shanghai University of Sport at 57-63% of the maximum heart rate (220-age) (17). The exercise frequency will be 30 minutes a day, 5 days a week, for a period of 12 months. The subject will wear a heart rate monitor during exercise to monitor exercise intensity. In the first 2-4 weeks, subjects will exercise for 15-30 minutes at a maximum heart rate of <57%. Then, the subject’s exercise intensity will gradually increase to 57-63% of the maximum heart rate, and the time will be increased to 30 minutes. In addition, the low-intensity exercise group will maintain the same health education and prohibition of high-purine diet as the control group.

#### Moderate-Intensity Exercise Group

Participants in moderate-intensity exercise group will be intervened at Shanghai University of Sport at 64-76% of maximum heart rate (220 - age) (17). The exercise frequency will be 30 minutes a day, 5 days a week, for a period of 12 months. The subject will wear a heart rate monitor during exercise to monitor exercise intensity. In the first 2-4 weeks, subjects will exercise for 15-30 minutes at 57-63% of their maximum heart rate. Then, the exercise intensity of the subjects will gradually increase to 64-76% of the maximum heart rate, and the time will be increased to 30 minutes. In addition, the moderate -intensity exercise group will maintain the same health education and prohibition of high-purine diet as the control group.

In the low-intensity exercise group and the moderate-intensity exercise group, all exercise sessions will be supervised by research staff. The exercise intensity, duration, heart rate and blood pressure before and after exercise, and adverse events during exercise will be recorded by study staff. The study participant retention is critically important and every effort will be made to increase participants’ adherence to their intervention and follow-up visit schedule. If the subject fails to participate in the study more than 3 times in a row, the researcher will call the subject to ask the reason and encourage the subject to continue participating in the study. During the research process, the researchers will also send personalized gifts to the participants to strengthen their enthusiasm.

### Outcome Assessment

Assessors will assess all primary and secondary outcomes at baseline, the 3rd, 6th, and 12th months. The evaluation process is shown in **Table 1**.

**Table 1:** Schedule for data collection, the process of the assessments per visits.

Measures	Baseline	Intervention phase		
Time (months)	-12-0	3	6	12
Informed consent	✓			
Random grouping	✓			
Demographics and Past medical history	✓			
Anthropometric parameters (body weight, waist circumference, hip circumference, BMI)	✓	✓	✓	✓
Physiological indicators (blood pressure, grip, vital capacity, maximum oxygen)	✓	✓	✓	✓
Biochemical indicators (blood lipid, blood sugar, liver enzyme, creatinine, and blood urea nitrogen)	✓	✓	✓	✓
Serum uric acid	✓	✓	✓	✓
Adverse events	✓	✓	✓	✓
Completion of the test form				✓

#### Primary Outcomes

The concentration of SUA, the amount of concentration change (mg/dL), the rate of change (%), and the proportion of patients with SUA concentration ≤7.0 mg/dL (male) or ≤6.0 mg/dL (female) from baseline to the 3rd, 6th, and 12th months will be observed. Detection method: The blood samples will be collected between 8:00 am and 10:00 am. Subjects should keep fasting for 12 hours, that is, eating and drinking after 8 o’clock the day before blood collection is prohibited. Each subject will collect 5 mL of blood. The blood sample will be centrifuged at 3500r/min for 15min. The obtained serum samples will be stored in a refrigerator at -80°C and tested by Zhongshan Hospital with Fudan University.

#### Secondary Outcomes

(1) Anthropometric parameters: body weight, waist circumference, hip circumference, BMI.
(2) Physiological indicators: blood pressure, grip, vital capacity, maximum oxygen.
(3) Biochemical indicators: blood lipid, blood sugar, liver enzyme, creatinine, and blood urea nitrogen.

#### Adverse Events

All adverse events reported during the study will be recorded on the case report form (CRF). In this trial, adverse events will be defined as any unfavorable and unexpected signs, symptoms, or diseases related to the intervention, such as falls, fractures, dizziness, high blood pressure, palpitations, chest pain, and low blood sugar. Researchers will ask the subjects about the adverse events they have experienced before the start of the training and record all the adverse events of the intervention. Participants will receive calls every week and ask about any adverse events experienced. Subjects will also be required to self-report any adverse events throughout the study protocol. A serious adverse event (SAE) will be notified to the lead investigator within 24 hours. The chief researcher is responsible for managing the safety report. Any adverse events related to the intervention will be reported to the ethics review committee of the Shanghai Institute of Physical Education. Although the committee and the author are from the same institution, the members of the ethics committee are independent of the investigators. None of these investigators are members of the ethics committee.

#### Mathematical Statistics

The data collected in the experiment will be statistically analyzed by SPSS 25.0, and the results will be expressed in the form of mean ± standard deviation. According to the specific values of each index, the Kolmogorov-Sminov test is used to determine whether it conforms to the normal distribution. If the index conforms to the normal distribution, the independent sample T-test is used to judge the difference between the two groups, and the paired sample T-test is used to judge the difference between each group before and after the intervention. If the index does not conform to the normal distribution, the Mann-Whitney test in the non-parametric test is used to judge the difference between the two groups, and the Wilcoxon test is used to judge the difference before and after the intervention in each group. Spearman correlation analysis is used to test the correlation of each index. P= 0-0.05 is set as the significance level.

## Discussion

Studies have shown that the improvement of SUA level helps reduce the risk of chronic disease, such as hypertension (28, 29), obesity (30), diabetes (31–33), insulin resistance (34, 35).

Several small clinical trials have evaluated the effect of short-term physical exercise on SUA in HUA patients. Shu Yuan and his colleagues reported that the SUA of a 45-day aerobic exercise program (1600-meter jogging) decreased by 10.5% (P <0.05) (27) This is consistent with the findings of J Strength Cond Res et al. They reported that patients with mild to moderate high blood pressure in men with 60-79% maximum heart rate reserve for 8 weeks of aerobic exercise, SUA reduced by 41.8% (P <0.05) (36). However, another study showed that 12 weeks of strength training increased the SUA of patients with type 2 diabetes (P <0.001) (37). The study by Li-Ling Huang et al. (20) showed that the SUA level of professional athletes was significantly increased after short-term high-intensity training (P <0.05). The mechanism may be reduced uric acid excretion after high-intensity exercise, which in turn leads to an increase in SUA. Therefore, low and medium exercise may be a suitable choice to improve HUA.

This study is a randomized, multi-center, parallel trial, which aims to explore the impact of low- and moderate-intensity exercise on HUA. The expected results of this study are (1): Low-intensity exercise (brisk walking for 150 min a week, 57-63% maximum heart rate) would have a significant decrease in SUA at 6 and 12 months for HUA patients (2). Compared with the control group and the low-intensity exercise group, the moderate-intensity exercise group (jogging for 150 min a week, 64-76% maximum heart rate) would have a greater reduction in SUA levels at 6 months and 12 months (3); Low-intensity exercise and moderate-intensity exercise can significantly improve body weight, waist circumference, fat composition, blood sugar, liver function, and other chronic diseases. These results can provide a basis for the current physical activity guidelines for HUA’s healthy lifestyle management.

Nevertheless, this experiment has some limitations. First of all, in this study, the control group only conducts lifestyle counseling, which would reduce the enthusiasm of the subjects and increase the difficulty of recruiting subjects. Second, the purpose of this study is to explore the independent effect of exercise on HUA. Therefore, we do not strictly control the intake of purine foods; only restrict the intake of high-purine foods in this study. Future clinical trials should study the effect of combined dietary intervention strategies on the long-term prognosis of HUA.

## Trial Status

At the time of submission of the manuscript, the status of the trial IS the pre-recruitment preparation stage. The recruitment of subjects is expected to be completed in July 2022.

## Ethics Statement

This study has been approved by the Ethical Review Committee of the Shanghai University of Sport (approval number: 102772020RT005). The publication of any potentially identifiable images or data contained in the article requires personal written informed consent. The research team will provide consultations for all subjects and their families to answer any research questions. Before signing the informed consent form, after the patients and their families fully understand the research process, our team members will organize the patients to sign the informed consent form or withdraw from the research. All subjects or their guardians will sign informed consent. Authors tend to submit research results to peer-reviewed journals or academic conferences for publication.

## Author Contributions

YH conceived the idea, supervised the research, was the guarantor, and prepared the first draft. RM and SG participated in the design. KK revised the manuscript. RW, TL, and HY conducted statistical calculations and provided research funding. All authors contributed to the article and approved the submitted version.

## Funding

Funding: the National Natural Science Foundation of China (31671242, 31971097), the National Key R&D Program of China (2020YFA0803800, 2018YFC1314700), and the Construction Project of High-Level Local Universities in Shanghai, China

## Conflict of Interest

The authors declare that the research was conducted in the absence of any commercial or financial relationships that could be construed as a potential conflict of interest.

## Publisher’s Note

All claims expressed in this article are solely those of the authors and do not necessarily represent those of their affiliated organizations, or those of the publisher, the editors and the reviewers. Any product that may be evaluated in this article, or claim that may be made by its manufacturer, is not guaranteed or endorsed by the publisher.
